# Institutionalizing Evidence-Informed Priority Setting for Universal Health Coverage: Lessons From Indonesia

**DOI:** 10.1177/0046958020924920

**Published:** 2020-06-08

**Authors:** Manushi Sharma, Yot Teerawattananon, Alia Luz, Ryan Li, Waranya Rattanavipapong, Saudamini Dabak

**Affiliations:** 1 Health Intervention and Technology Assessment Program, Ministry of Public Health, Thailand; 2 Saw Swee Hock School of Public Health, National University of Singapore, Singapore; 3 Imperial College London, United Kingdom

**Keywords:** universal health insurance, technology assessment, biomedical, Indonesia, health policy, health resources, investments, institutionalization

## Abstract

Planning and administering Universal Health Coverage (UHC) policies involve complex and critical decisions, especially in resource-scarce and densely populated settings such as Indonesia. Increasing investments alone do not ensure success and sustainability of UHC, and defining priorities is imperative. In 2013, Indonesia formally embarked on its journey of institutionalizing priority setting with technical assistance from the International Decision Support Initiative (iDSI), which is a global network of organizations in pursuit of evidence-based priority setting. This article provides a perspective for countries in pursuit of institutionalization of evidence-informed policy setting systems and sheds light on the factors conducive to the development of health technology assessment (HTA). It explores the main actors and the context of priority setting in Indonesia and articulates strategies and key outcomes and impact using the theory of change (ToC).

**What do we already know about this topic?**
Tools for evidence-based priority setting have gained popularity, especially for developing countries rolling-out universal health coverage (UHC). Financial sustainability of the scheme is of utmost concern. This article adds to the available material on evidence-informed priority-setting methods, i.e., institutionalization of HTA in Indonesia.
**How does your research contribute to the field?**
This article provides a perspective for countries in pursuit of institutionalization of evidence-informed policy systems and sheds light on the factors conducive to the development of HTA. It explores the main actors and the context of priority setting in Indonesia and articulates strategies and key outcomes and impact using the theory of change (ToC).
**What are your research’s implications toward theory, practice, or policy?**
This research has implications for both practice and policy. The Indonesia case resonates with low- and middle-income countries (LMICs) as they graduate from foreign aid and are in the pursuit of delivering UHC but are facing challenges related to health financing and priority setting. Next, evaluation of capacity-building initiatives at the institutional and system levels is very complex as the intangible effects such as social and individual transformation are difficult to measure by the most adopted evaluation methods. This study attempts to bridge this knowledge gap.

## Introduction

Indonesia formally launched the universal health coverage (UHC) scheme under the banner of Jaminan Kesehatan Nasional (JKN) in 2014,^1^ which is operated by the Social Security Administrator, Badan Penyelenggara Jaminan Sosial (BPJS) Kesehatan. JKN is one of the largest single-payer health insurance (SHI) schemes in the world.^1,2^ By October 2018, JKN covered 203 million people, and by 2017, it had paid out US$20.15 billion (US$ purchasing power parity [PPP]) for 223.4 million consultations for both primary and advanced treatments.^3^ However, the geographic, human, and economic diversity of the world’s fourth most populous nation poses numerous challenges to the JKN. Foremost concerns for the BPJS are JKN’s financial stability and its ability to meet the health needs,^4^ attributed to the following factors. First, there is a high insurance premium, which makes some services inaccessible, eg, cancer treatment, dialysis, and maternal health services.^3^ Second, even though in the past 10 years Indonesia has increased its investment in health, it remains small on per capita basis in comparison with neighboring countries such as Malaysia, Thailand, and Singapore.^5^ And, third, the health benefits package (HBP) and the list of essential medicines (Formularium Nasional, FORNAS) reimbursed under the JKN is very generous, even by high-income country standards.^4^ The expanding coverage of JKN, the population’s growing health awareness, changes in health-seeking behavior, and decreasing external assistance in health have placed financial pressure on the resources resulting in a large financial deficit with a medical claim ratio of 115% in 2014,^4^ necessitating the use of evidence-informed priority-setting (EIPS) tools. To address the need for an EIPS, the Ministry of Health formally established the Health Technology Assessment Committee (InaHTAC) through a decree in 2013^6^ to function as a focal point for all health technology assessment (HTA) activities that would ultimately support the decisions of BPJS. This development fueled the HTA movement in Indonesia; however, a general lack of capacity in EIPS both in the production of evidence and in acceptability of rational priority setting as a policy solution among high-level policy makers,^7^ paved the path for collaboration between the Ministry of Health (MoH) in Indonesia and the International Decision Support Initiative (iDSI).^8^ iDSI’s mission is to guide decision-makers to effective and efficient healthcare resource allocation strategies for improving health outcomes of the population. Over half a decade, 2 of iDSI’s core partners led this collaboration, the Health Intervention Technology Assessment Program (HITAP) and Imperial College London (formerly National Institute for Care Excellence [NICE] International).

**Box 1.** Timeline of UHC and Priority-Setting Development in Indonesia.

• Prior to 2011, various insurance schemes provided the majority of health coverage in Indonesia. • In 2011, a single security system management agency—Badan Penyelenggara Jaminan Sosial was established. • Subsequently, in 2012, the national government issued a Jakarta Health Card (JKS) which is essentially considered as a pilot for the UHC initiative in Indonesia. • In 2013, through a presidential regulation 12/2013^6^, the government mandated the use of health technology assessments and subsequently the Health Technology Assessment Committee (InaHTAC) was established. • Finally, on January 1, 2014, the UHC was formally launched with the target of attaining 100% coverage by 2019.

*Source.* Adapted from Itad Report.

*Note.* UHC = universal health coverage.

This article provides an account of strategies undertaken by iDSI in Indonesia and describes the impact using the iDSI Theory of Change (ToC).^9^ The ToC sets out the causal steps and preconditions for translating iDSI support into the institutionalization and capacity-strengthening required for EIPS at the country level, leading to better resource allocation decisions with improved population health outcomes and impact.^10^ It is a nonlinear framework underpinning iDSI’s monitoring and evaluation, strategic planning of activities, communication of impact, and learning as a network. In this article, we describe our observations from Indonesia using the first 3 components of the ToC, namely, effective partnerships, institutionalizing EIPS at the country level, and making better decisions. We adapted and applied a stakeholder analysis tool, developed by Vlad,^11^ to describe the priority-setting landscape in Indonesia and to identify the roles and positioning of the stakeholders that partnered with iDSI in different stages and capacities. To build a holistic narrative, we collected the information and data through multiple sources: a review of evaluations conducted by external evaluators,^8^,^12^ mission reports (logs of activities and outputs maintained by iDSI project managers), relevant academic and gray literature, and discussions with stakeholders. With this study, the authors aim to supplement the existing literature on the institutionalization of EIPS through HTA’s in developing countries^13-15^; we describe our experience and key learnings from supporting Indonesia in its HTA journey, reflecting on the nuts-and-bolts of institutionalizing EIPS and its implications for future HTA development in the country.

## iDSI in Action—ToC

**Figure 1.**
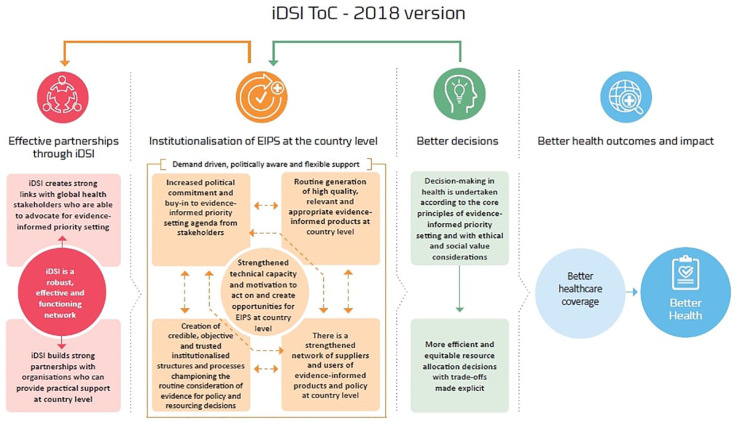
iDSI ToC. *Note.* iDSI = International Decision Support Initiative; ToC = theory of change.

To better understand how, why, and to what extent the change is attributed to iDSI engagement, we use the ToC framework developed by Itad in 2015 and refined in 2018 (Figure 1) in consultation with iDSI partners.^9^ The framework has 4 pillars:

1. *Effective partnerships through iDSI*: This pillar emphasizes the importance of establishing strong links with and between global and local partners such that iDSI is able to mobilize the right kinds of capacity in providing technical assistance, building trust and collaboration between individuals and organizations in-country where such links previously did not exist (eg, connecting researchers directly with policymakers) and foster an enabling environment for EIPS and HTA, and allowing for the emergence of champions.
2. *Institutionalization of EIPS at country level*: Through iDSI’s provision of politically aware, demand-driven, and flexible support, capacities to generate and use evidence are built and strengthened. Furthermore, the individuals and organizations engaging in EIPS increase in number and have stronger, more connected networks. Through this, EIPS becomes the norm (and thus institutionalized) through the establishment of processes and structures. This arm encompasses 5 interdependent strategies involving all stakeholders, explained in the next section.
3. *Better decisions*: Resource allocation and purchasing decisions in health (eg, HBP coverage decisions or pricing decisions for a particular health intervention) are *procedurally* in keeping with the principles of EIPS, eg, undertaken transparently, independently and consideration of relevant health economic and contextual evidence. As such, the decisions result in investment in, eg, more cost-effective or equitable interventions.
4. *Better health outcomes and impact*: Should ensue if the first 3 pillars are satisfied, subject to implementation constraints at the policy and clinical levels. Note that downstream population health impact is typically expected only to be realized beyond the time horizon of typical iDSI support. However, it is possible to model the potential impact *ex-ante*.^16^

## Results

This section is structured in congruence with the ToC: “Effective Partnerships Through iDSI” subsection explains regional and global partnerships that were forged to reinforce a favorable ecosystem for HTA; “Institutionalization of EIPS” subsection explains the specific activities that were undertaken to build the capacity of the users and consumers of HTA over the period of 5 years; “Better Decisions” subsection explains the decisions that were taken for the JKN in Indonesia.

### Effective Partnerships Through iDSI

Regional and global partnerships are central to promote EIPS, knowledge management, and capacity development. Connecting the existing Indonesian and international networks allowed the Indonesian stakeholders to circumvent issues that currently inhibit the EIPS and knowledge-management initiatives. Over 5 years, 10 technical training workshops, 2 high-level meetings, topic selection meetings, and stakeholder consultation meetings,^17^ and participation in international policy forums like the Prince Mahidol Award Conference and the HTAsiaLink Annual Conference were supported.

A local network of producers was convened to ensure the EIPS agenda is locally driven. iDSI’s technical support in collaboration with international agencies proved to be beneficial in setting up the local network, as these agencies are influential regionally.

Partners like Access and Delivery Partnerships (ADP) and the World Health Organization (WHO) helped by identifying and providing local champions across the country with financial sustenance to kick-start the EIPS initiative, which gradually gathered momentum and was able to earn the support of policymakers.

Table 1 describes the stakeholders who are associated with Indonesia’s HTA journey, detailing their role in the health sector as well as in priority-setting and the strategies that were employed to forge effective partnerships, adapting the tool by Vlad. The information in this table has been taken from the field and scoping reports.^18-26^ The organizations have been categorized based on their roles as consumers, producers, and supporters.

**Table 1.** Stakeholders in Priority Setting in Indonesia (2014-2019).

Institution	Type	Category	Role and positioning of priority setting and HTA	Strategies
BPJS	Government	Consumer of HTA evidence	● National UHC payer, supportive of EIPS ● The administrator of JKN, which was allotted IDR 71.6 trillion in 2016 (National Health Accounts—Indonesia, 2016). The BPJS has authority to make decisions regarding what goes in and out of the health benefits package and the list of essential medicines or the FORNAS. In 2013, it was mandated by law to incorporate evidence in its decisions for resource allocation^6^ ● Commissions HTA studies on priority topics ● Composed of mainly high-level policymakers who, although supportive of HTA, are unaware of its applications in the evaluation of policy, interventions, price negotiations	● Initial engagement was targeted to gain buy-in from high-level personnel to make the broader institutional setting more receptive for EIPS; activities include ○ Developing the capacity of top leadership through workshops^21,22^ and participation in international policy forums like PMAC and the HTAsiaLink conference^27^ ○ Several communications materials were designed to make the technical information palatable for the high-level policy makers^27–34^ ○ Last, engaging with the top-order officials in the evidence generation process, ie, topic nomination,^26^ regular consultations and deliberations, to results dissemination
InaHTAC	Government	Producer of HTA	● Advisory committee established through a presidential decree ● Focal point for coordinating all HTA activities in Indonesia ● This committee, including mostly clinicians and academics, was formed in response to a legislative decree^6^ ● Strong support for EIPS in 2014, but lack of technical know-how in conducting HTA ● Does not have any decision-making authority; however, they provide authoritative advice/policy recommendations to the health provider	● With the help of iDSI, a hub-and-spoke model was conceived. (The InaHTAC as the hub and universities and other think-tanks as the spokes) ● Local network of HTA producers was reinforced by identifying and outsourcing priority topics to universities (UI, University of Gadjah Mada, University of Padjajaran) and other local organizations such as the *Litbangkes*^13-21^ ● iDSI leveraged its global network, with experts such as health economists and experienced policy analysts brought in to provide recommendations, training and guidance to the local researchers ● Knowledge exchange and awareness forums were also organized to develop the capacity of senior InaHTAC members
P2JK Center of Health Financing and Insurance	Government	Supporter/producer of HTA	● The P2JK regulates the social health insurance scheme and health financing in general. It has 3 main subunits—economic evaluation (iDSI’s main point of contact), health financing, and tariff calculation for the Indonesian Case-Based Groups ● Secretariat to the InaHTAC. Some of the researchers from this team led HTA studies. ● Supports EIPS. However, limited technical know-how of conducting HTA	● P2JK team was involved closely in all the studies
NIHRD	Government	Producer of HTA	● Research Institute ● Support EIPS: they perform research related to health but lack the technical know-how of conducting economic evaluations and HTA research	● NIHRD also contributed to the local HTA capacity in the country by organizing capacity-building workshops^35^ ● Several capacity-building workshops were held to enhance the knowledge of this team^13–21^ ● Scholarships to foster long-term capacity and influence the HTA ecosystem were provided^36^
UI	Academic institution	Producer of HTA	● Supports EIPS: One of the premier institutions which generates evidence for policy in Indonesia ● Some senior members are a part of the InaHTAC, and young researchers conduct HTA studies ● In late 2015, the university hired skilled personnel in the Center of Health Economics Policy Studies (CHEPS) to conduct HTA research	● Rigorous capacity-building activities included in in-country technical workshops, web-based consultations, internships at HITAP^17,18,20,23^
UGM	Academic institution	Producer of HTA	● Supports EIPS: but lacks the technical know-how of conducting HTA-specific research ● In late 2015, put together a dedicated team to conduct HTAs and economic evaluations	● Rigorous capacity-building activities included in-country technical workshops, web-based consultations, internships at HITAP^17,18,20,23^
UNPAD	Academic institution	Producer of HTA	● Supports EIPS ● This group comprises working-level stakeholders, young researchers who perform HTA ● More recently, a team from UNPAD was commissioned by InaHTAC to conduct an HTA study	● Capacity building through workshops^37^ and consultations
ITAGI	Government	Consumer/producer of HTA	● Vaccine advisory committee ● Supports EIPS: provides recommendations to the MoH related to vaccine-related matters eager to learn HTA ● Have established a health-economics working group	● Capacity-building activities include a workshop on vaccine economics^18^ and study visit to HITAP for guidance on economic evaluation of rotavirus vaccine
WHO	Multilateral agency	Supporters of HTA and donors	● Supporters of EIPS ● In the initial phase, WHO Indonesia office served as the main point of contact for iDSI and provided financial support for one of the early HTA pilots (package of essential noncommunicable program)	● With over 25 UN agencies operating in Indonesia, WHO is one of the lead agencies and has convening power bringing together stakeholders within the MoH and others such as universities ● WHO’s support helped iDSI connect BPJS, PPJK and ITAGI; they also provided financial support for HTA studies and other capacity-building initiatives
AIPHSS	Multilateral agency	Supporters of HTA and donors	● Supporters of EIPS ● Providers of financial support for capacity-building activities from 2014 to 2016	Similar to WHO, this group of stakeholders provided financial support for the studies and other capacity-building activities
ADP	Multilateral agency	Supporters of HTA and donors	● Supporters of EIPS ● ADP is a key partner in Indonesia ● Through ADP’s financial support, HITAP was able to support the local team who were reviewing laws and regulations of off-label medicines in Indonesia ● ADP through PATH supported various capacity-building initiatives and the development of the national HTA plan in the country^38^	● ADP support was crucial and provided the required boost to the InaHTAC in furthering its intent to develop a system for EIPS ● One of the main outcomes was the first national HTA guidelines for Indonesia
International Decision Support Initiative	International technical support partners	Technical partners	● Delivery partner and supporter of EIPS. ● Global network of health, policy, and economics experts. Works with countries to achieve UHC through good-value for money investments ● Main partners—HITAP and Imperial College, London (former National Institute for Care Excellence International)	● iDSI’s end-to-end support covered several activities such as, topic selection, stakeholder consultations, capacity building, developing relevant knowledge products, networking activities which supported the HTA movement to take roots and spread. Outcomes include: ○ In 2016-2017, 4 studies recommended potential savings of US$31.9 million for the BPJS ○ Off-label drugs (sildenafil) were registered under the JKN^39^ ○ Other outcomes such as the development of HTA roadmap and guidelines, high-level meetings, linking research on EQ-5D-5L value set for Indonesia to policy processes and methods for HTA

*Note.* HTA = health technology assessment; BPJS = Badan Penyelenggara Jaminan Sosial; UHC = universal health coverage; EIPS = evidence-informed priority-setting; JKN = Jaminan Kesehatan Nasional; PMAC = Prince Mahidol Award Conference; InaHTAC = Health Technology Assessment Committee; iDSI = International Decision Support Initiative; UI = University of Indonesia; NIHRD = National Institute of Health Research and Development; HITAP = Health Intervention and Technology Assessment Program; UGM = University of Gadja Mada; UNPAD = University of Padjadjaran; ITAGI = Immunization Technical Advisory Group in Indonesia; MoH = Ministry of Health; WHO = World Health Organization; UN = United Nations; AIPHSS = Australian Indonesian Partnership for Health Systems Strengthening; ADP = Access and Delivery Partnerships.

Consumers are stakeholders who interpret and use the results of HTA to feed into healthcare decisions, eg, BPJS. Producers are stakeholders who conduct policy-relevant research, eg, academics and researchers. Supporters are those who facilitate the priority-setting agenda by providing resources—both financial and technical—linking with networks, and other types of support, such as that provided by, eg, ADP, WHO, and the iDSI. Other important stakeholders are patient associations, clinical associations, private pharmaceutical firms who wield influence in health care decision making. iDSI did not directly interface with these stakeholders, and so they are not discussed in this piece. Although this table is described under this section, the strategies discussed here are closely related to the other 3 arms of the ToC.

### Institutionalization of EIPS

Over the years, the UHC has become an electoral asset in Indonesia, gradually moving ranks on the political agenda, which translated in increased investment in health.^5^ However, improved investment does not necessarily deliver better health outcomes if the value of this financing is low. To mitigate the risks associated with low-value investments, the president’s office established the InaHTAC, demonstrating an institutional commitment to EIPS to some extent. Albeit the limited technical capacity within InaHTAC, a general scarcity of technically sound professionals in the broader health system to undertake economic evaluation and other HTA production activities acted as barriers to the success of the InaHTAC.^40^ Furthermore, there was a limited budgetary commitment to EIPS with small and scattered domestic financial support for local agencies to conduct HTA studies. Similarly, on the demand side, in the former years (2014-2016), insufficient awareness and knowledge among policymakers and payers (especially high-level decision-makers) about the benefits and uses of HTA in resource allocation and purchasing presented a barrier to the uptake of HTA and its translation into evidence-informed policies.

iDSI engaged in continued and consistent efforts, with the Indonesian counterparts which led to an increase in awareness regarding HTA as a tool for priority setting and strengthened the link of research to policy. Main activities include working with key Indonesia stakeholders such as InaHTAC, in coordination with other international partners such as the WHO, ADP on activities such as HTA studies, development of national HTA guidelines, capacity-building exercises, knowledge exchange forums.

iDSI targeted technical capacity of both users and producers of HTA to achieve institutionalization of EIPS. In 2014, HITAP supported the local research in the economic evaluation of the WHOs Package of Essential medicines for Noncommunicable diseases (PEN) program. The purpose of this study was 2-fold, to demonstrate the relevance and applicability of HTA to achieve UHC and to build technical capacity by providing targeted, hands-on training. Engagement with high-level dignitaries throughout the process helped in gaining their support. While other evaluations of renal dialysis and PEN were ongoing, the evaluation of sildenafil which is an off-label medicine used for pulmonary arterial hypertension (PAH) was requested by high-level clinicians from the association of pulmonologist to the Center of Health Financing and Risk Protection (Pusat Pembiayaan Jaminan Kesehatan).^30^ The evaluation proved that sildenafil was cost-effective. Contrary to the prevailing scenario where the JKN did not allow the use of off-label medicines due to legal constraints, the study revealed that countries like Thailand and Australia registered off-label medicines under the UHC. Sildenafil was inaccessible to patients with PAH in Indonesia, remaining unregistered for this indication under the JKN^41^ (Table 2). Post completion of the research project, dissemination meetings were held with all stakeholders, including but not limited to patient groups, clinicians, policymakers, and pharmaceutical companies. Although many stakeholders called the results “controversial,”^26^ the MoH decided to include sildenafil in the JKN benefits package,^39^ and the medicine registration process was expedited, allowing for the consideration of safe and cost-effective off-label medicines for other ailments under the JKN benefits package. Among other activities (evaluation of PEN program, renal dialysis, and development of process and methods guidelines) that were ongoing in phase 1, the study on “off-label” medicines gained much attention and traction as it was a topic of national and global interest. Consequently, this evoked the interest of policymakers in other activities of the InaHTAC. More HTA studies were pursued in the following years based on the request from BPJS (Table 2). These studies catered to the immediate demand of HTA in Indonesia, and simultaneously, efforts were made to streamline the EIPS process and structures.

**Table 2.** HTA Studies in Indonesia (2014-2018).

No	Year	Funder	Topic	Rationale for topic selection	Description	Recommendations and potential impact
1	2014-2015	WHO	*Economic evaluation of screening and treatment for diabetes and hypertension (PEN program)* ^42^	After 3 years of PEN policy implementation, the effectiveness and impacts of the program were unknown. Thus, on the request of the Ministry of Health, this topic was chosen.^42^	The existing PEN program was an adaptation of the WHO PEN guidelines for low- and middle-income countries. This quantitative evaluation examined the cost-effectiveness of the PEN program compared with a “no screening” policy. For further streamlining the policy, this assessment also explored the cost and outcomes of modifying the current PEN program through changes in the target population and screening strategies.	With a small tweak in the current policy of selectively screening the high-risk population, the coverage could be increased from 28% to 63%.
2	2015-2016	Ministry of Health, Indonesia and the AIPHSS under the Department of Foreign Affairs Trade, Australia	*Economic evaluation of policy options for dialysis treatment for end-stage renal disease patients under UHC in Indonesia* ^28^	It is estimated that only 53% of patients have access to dialysis, with a majority being administered the HD, despite PD being the cheaper option. This investment was the second largest expense incurred by the BPJS.	This economic evaluation compared no dialysis and 2 dialysis policy options, ie, HD-first (current approach) and PD; both the options can be reimbursed under the UHC in Indonesia.	The PD-first policy was found to be more cost-effective. Potential savings of IDR 1.3 trillion for the UHC provider from switching to PD from HD.
3	2016-2017	Ministry of Health, Indonesia and the AIPHSS under the Department of Foreign Affairs Trade, Australia	*Economic evaluation of sildenafil for the treatment of PAH in Indonesia* ^30^	Several countries allow for the use of sildenafil for PAH, given its clinical efficacy and cost-effectiveness. However, as an off-label medicine, it is not prescribed under the UHC. Registration of off-label sildenafil could be a value proposition for the BPJS. Thus, on the request of the PAH association of Indonesia, its cost-effectiveness was assessed.	This study explores the cost-effectiveness and budget impact of adopting sildenafil to the benefits package for the indication of PAH compared with beraprost.	It was found that sildenafil was cost-effective compared with beraprost. Following this study, the sildenafil was registered for use under the UHC.^40^
4	2016-2017	PATH through the ADP	*Review of laws, regulations, and uses of off-label drugs in Indonesia.* ^41^	This study was conducted to explore the current situation of the off-label use of medicines in Indonesia.	It describes the advantages and disadvantages of health, economic, and ethical impacts along with policy recommendations.	Recommendations for reimbursement of priority off-label medicine with proven clinical benefits under the UHC scheme.
5	2017-2018	National UHC payer (BPJS)	*Nilotinib for CML treatment* ^33^	Nilotinib should be used only when the patient is resistant to first-line treatment (imatinib), but the rate of nilotinib use is twice as high. Also, this drug is a budget-burner for the BPJS.	Qualitative study on irrational nilotinib use and treatment patterns of CML under UHC in Indonesia.	A total amount of US$0.5 million is saved in the elimination of the irrational nilotinib use.
6	2017 -2018	National UHC payer (BPJS)	*A systematic review of the use of Insulin analogue for type 2 diabetes* ^34^	Analogue insulin is widely used in Indonesia despite international recommendations that advocate the use of less costly human insulin, the first generation of man-made insulin, as a first-line treatment. This prescription pattern places an immense burden on the BPJS.	A systematic review of the use of insulin analogues, which is listed in FORNAS compared with human insulin, was conducted.	If the price is negotiated and the use is regulated, ie, similar to the implementation in other countries like Thailand, human insulin can provide savings. The UHC provider can save approximately US$9 million annually.
7	2017-2018	National UHC payer (BPJS)	*Economic evaluation of cetuximab for metastatic coleo-rectal cancer patients* ^31^	Cetuximab is a cost-ineffective drug and is not reimbursed even in developed countries. Indonesia, however, spends a substantial amount of health budget in reimbursing this drug. Thus, on the request of BPJS, this topic was chosen	This study compares the following interventions—FOLFIRI, FOLFOX, cetuximab plus FOLFIRI, and, cetuximab plus FOLFOX. The cost-utility analysis was conducted from a societal perspective using a Markov model. The budget impact was analyzed from the payer perspective.	This study found that using cetuximab in combination with chemotherapy is not cost-effective when compared with chemotherapy alone. Also, cetuximab is being used for indications not listed in the Indonesian clinical guidelines. Following the policy recommendation from the study, cetuximab had been removed from the national formulary, with potential savings of US$9 million per annum for the UHC provider. However, due to resistance from the clinicians this decision was reversed.
8	2017-2018	National UHC payer (BPJS)	*Bevacizumab for mCRC* ^32^	Similar to cetuximab, bevacizumab imposes a huge financial burden on the BPJS. Thus, the Health Technology Assessment Committee was asked to pursue the assessment of this drug.	Bevacizumab is reimbursed under the UHC program for mCRC treatment. This study evaluates the use of bevacizumab in combination with chemotherapy compared with chemotherapy alone.	The findings suggest that adding bevacizumab to chemotherapy is not cost-effective. Bevacizumab had been removed from the national formulary and this move had the potential of saving the BPJS an amount of US$14 million annually. However, due to resistance from clinicians this decision was reversed.

*Note.* HTA = health technology assessment; WHO = World Health Organization; PEN = package of essential noncommunicable; AIPHSS = Australian Indonesian Partnership for Health Systems Strengthening; UHC = universal health coverage; HD = hemodialysis; PD = peritoneal dialysis; BPJS = Badan Penyelenggara Jaminan Sosial; PAH = pulmonary arterial hypertension; ADP = Access and Delivery Partnership; CML = chronic myeloid leukemia; mCRC = metastatic colorectal cancer.

Institutionalization of an EIPS in a country is a continuous process. Inclusive and transparent processes with legislative arrangements are required to ensure compliance from all stakeholders (patients, health professionals, providers, manufacturers, etc). iDSI leveraged its network and experts from NICE international and HITAP and put in collective efforts to support the development of the national HTA guidelines or the “ground rules” to ensure consistent, transparent, and legitimate MoH-endorsed processes throughout the country.^43^ The InaHTAC, with help from experts at NICE International, developed an HTA roadmap.^44^ The roadmap is the “blueprint” of the HTA process and mechanism in Indonesia. Several consultation meetings, both regional and bilateral, were held to share the success, failures, and lessons learned by HITAP and NICE International to aid Indonesian counterparts in making a bespoke HTA strategy to fit the local context and policy needs.

In addition, to foster institutional capacity, HITAP hosted Indonesian delegates as interns and provided a scholarship to a researcher to pursue higher education in economic evaluation.^33^ The program was comprehensive, with activities including observing price negotiation meetings at the Thai National Health Security Office (NHSO), the BPJS equivalent, as well as mentorship and consultations with experts from HITAP and Mahidol University. iDSI linked researchers at the UNPAD who had developed the EQ-5D-5L value set which was used to develop, QALY, the measure of health outcome used in cost-effectiveness research for Indonesia with the InaHTAC. All these activities led to a strengthened network of suppliers of evidence-informed products and policy at country level.

Significant strides were taken in capacity building for HTA in both supply and demand sides against the baseline capacity of the country which was very low.^8, 12^ The outcomes of technical capacity building are tangible as compared with institutional capacity building mainly due to the complex network of organizations/stakeholders that wield influence over priority setting in Indonesia and are beyond the control of the InaHTAC. The local network is still evolving, and in future, efforts should be directed toward strengthening the network of users to influence the policy aspects of EIPS.

### Better Decisions

“The good news is that evidence can matter. The bad news is that it often does not.”

—Julius Court

The InaHTAC, through iDSI’s support, has made an impact in streamlining Indonesia’s efforts to achieve UHC by providing contextual evidence (as also summarized in Table 2).^8,12^ The registration of sildenafil for the treatment of PAH is one of the most apparent and significant impacts of this collaboration.^39^ However, policymaking is inherently a political process, and many factors jostle with evidence to take the limelight in policy formation. The InaHTAC does not have any decision-making authority. Nonetheless, systematic efforts have created a conducive environment for the uptake of evidence into policy. The result dissemination events involved several local stakeholders and created awareness and avenues for collaboration. Topics of interest were pursued independently. The renal dialysis study did not result in direct policy change; however, in 2017, an independent knowledge-enhancing activity was organized by the local authorities for the practitioners in Bali.^45^ More recently, cetuximab and bevacizumab were removed from the HBP following the recommendations of the studies^46^ but were revoked soon after due to critique and refusal from the patient organizations and clinicians. Together, removal of both the drugs would have saved US$23 million for the BPJS. This is an important lesson that HTA is part of the broader health system in which several stakeholders operate, which is also an obstacle for the translation of evidence into policy. Despite this, the mere fact that the HTA studies were used to make an informed policy decision arguably represents progress.

## Discussion

The Indonesia case provides valuable lessons for the countries on the quest of institutionalizing EIPS for attaining and sustaining UHC. The HTA movement in Indonesia has established a solid foundation, courtesy of the establishment of the InaHTAC, an independent body performing HTA research to provide contextual evidence through systematic processes.^40^ Several factors impede the progress of institutionalization of EIPS and HTA in the Indonesian health system. The establishment of InaHTAC through a decree demonstrated early foundational support and institutional commitment of policymakers for EIPS. However, HTA has not been used comprehensively into policy decisions partly because of a general lack of capacity among health policymakers to absorb HTA and its applications in improving resource allocation and purchasing. iDSI has played an important role in facilitating engagement, knowledge transfer, and capacity building both within and between suppliers and users of HTA. However, in order for HTA to be sustained, the agenda has to be led locally. To a certain extent, conditions have improved since iDSI first liaised with the MoH in 2013, with BPJS demonstrating budgetary commitment and stepping up as a funder for HTA studies toward the latter part of iDSI’s engagement in 2016-2017 as seen in Table 2. This includes the decision to delist cetuximab and bevacizumab. Unfortunately, even though delisting the 2 drugs would have immensely relieved the BPJS from financial agony, the decision was reversed, and this underscores the critical need for buy-in and effective collaboration from all stakeholders in order for the EIPS to function. Next, iDSI’s capacity-building activities were conducive in creating local capacity (producers of HTA) for performing economic evaluations and HTAs, but the absence of a robust health information infrastructure is unfavorable, and HTA research can be simplified with easy procurement of quality data. Finally, a decentralized healthcare system and the presence of multiple stakeholders add another level of intricacy and act as a barrier for translation of research into policy.

The ToC proved to be beneficial not only in helping iDSI articulate and administer its in-country support but also in monitoring and evaluating impact in terms of strengthening institutions for EIPS and improving the quality and value of health resource allocation decisions. The ToC can be a powerful tool to communicate and capture the complexity of an intervention like the iDSI country support in Indonesia.

HTA initiatives implemented in other settings differ in their responsibilities, structure, and relationship to final policy decisions. Studies conducted in Thailand, India, and other developing countries^14,47,48^ show that there is no one-size-fits-all approach for the institutionalization of HTA. Each country has its distinct set of features that acts as a barrier or enabler for the institutionalization of HTA. In Indonesia, some factors that were enablers for EIPS are the establishment of InaHTAC by a presidential decree, endorsement of the HTA guidelines by the MOPH (which detail the HTA process for Indonesia, and creation of a local network of HTA producers). However, certain factors such as limited capacity of users and other stakeholders such as clinicians and patients organizations to comprehend the benefits of EIPS, weak stakeholder engagement, no mandate for inclusion of evidence pertaining to cost-effectiveness in decision making, unclear policy processes and procedures, and weak data infrastructure are impediments to the institutionalization of HTA and EIPS.

This study is one of its kind in the Indonesian setting. This article is authored by iDSI network members directly involved in delivering and may, therefore, be subject to self-reporting bias; however, our findings do not contradict the conclusions of a learning review of iDSI’s Indonesia engagement undertaken by an independent evaluator.^8,12^ Also, iDSI’s engagement in Indonesia did not include contact with patient groups, pharmaceutical companies, other ministries, eg, Ministry of Finance, the media, and other stakeholder groups that have an impact in the development of the EIPS. Further exploration of these perspectives would be useful as Indonesia continues its UHC journey. It is also worth exploring quantitatively the impact of HTA in the future, once the implementation of the policies has been underway.
